# Does the appearance of the cutaneous scar after cesarean section reflect the residual myometrial thickness?

**DOI:** 10.1007/s00404-020-05943-2

**Published:** 2021-01-07

**Authors:** Ammar Al Naimi, Niki Mouzakiti, Carmen Eißmann, Frank Louwen, Franz Bahlmann

**Affiliations:** 1 Department of Obstetrics and Gynecology, Dr. Senckenberg Foundation, Buergerhospital, Nibelungenallee 37-41, 60318 Frankfurt am Main, Hessen Germany; 2 Department of Obstetrics and Gynecology, University Hospital, Goethe University Frankfurt am Main, Theodor-Stern-Kai 7, Frankfurt am Main, 60590 Hessen Germany

**Keywords:** Manchester scar scale, Cesarean section, Ultrasound, Residual myometrial thickness

## Abstract

**Purpose:**

The aim of this study is to utilize the Manchester scar scale (MSS) and ultrasound in investigating the association between uterine wall defects and cutaneous scar characteristics after cesarean section (CS).

**Methods:**

This is a prospective cohort study. The degree of myometrial loss was quantified by calculating a residual myometrial thickness (RMT) ratio as a percentage of RMT to the pre-cesarean anterior uterine wall thickness. Cutaneous scar assessment was performed according to the MSS. Spearman’s correlation and the Kruskal–Wallis test with a cut-off value of *p* < 0.05 were used for statistical analysis.

**Results:**

Two hundred forty seven women, of which 2.4% had an Asian, 3.6% an Afro-American, 82% a Caucasian and 12% a Mediterranean background, were recruited. The RMT ratio ranged between 11.9 and 100% with a median of 55.8% and an average of 56%. MSS scores ranged from 4 to 13 with a median of 5 and an average of 6. Spearman’s correlation between MSS and RMT ratio show a rho of − 0.01 with a *p* value of 0.8. The correlation between MSS and RMT ratio within the four ethnical groups showed a *p* value between 0.3 and 0.8 and a rho between 0.8 and − 0.8. The Kruskal–Wallis test showed an eta^2^ of 0.13 and a *p* value of 0.0002 for the effect of ethnicity on MSS and an eta^2^ of 0.009 and a *p* value of 0.68 for the effect of ethnicity on the RMT ratio.

**Conclusion:**

CS laparotomy scars heal differently between ethnical groups, but generally with satisfying results. Ethnicity does not affect myometrial healing and scar appearance does not reflect myometrial healing after CS. Thus, separate uterine sonographic assessment is recommended.

## Introduction

Due to the worldwide increase of cesarean section (CS) rate to about 30% in developed countries, CS with a suprapubic (Pfannenstiel-Kerr) incision has become the most common abdominal surgery for women [1]. These laparotomy scars heal relatively well, and their anatomical position makes them easy to hide if healing is not cosmetically satisfactory. Therefore, for a surprisingly long time obstetricians tended to ignore the importance of this scarring [2]. Deformed scarring has psychological effects and can lead to social dysfunction, communication hurdles and self-confidence issues. In addition to this distress, scarring can cause physical discomfort, pain, and pruritus [3]. With increasing focus on wound healing, several tools have been devised to objectively describe scar healing either quantitatively or qualitatively, and these tools have been utilized for assessing cutaneous scarring after a CS [4]. One of these assessment tools that are popular for objectively assessing different cutaneous scars is the Manchester Scar Scale (MSS) [5].

Uterine wall defects at the CS site are common [6], and several studies showed the association between these defects and gynecological symptoms such as spotting and chronic pelvic pain [7, 8]. Vaginal ultrasound represents the gold standard for assessing uterine wall defects after CS [9]. The aim of this study is to utilize ultrasound and MSS in investigating the association between the severity of uterine wall defects and the characteristics of the cutaneous scars after CS.

## Materials and methods

**Fig. 1:**
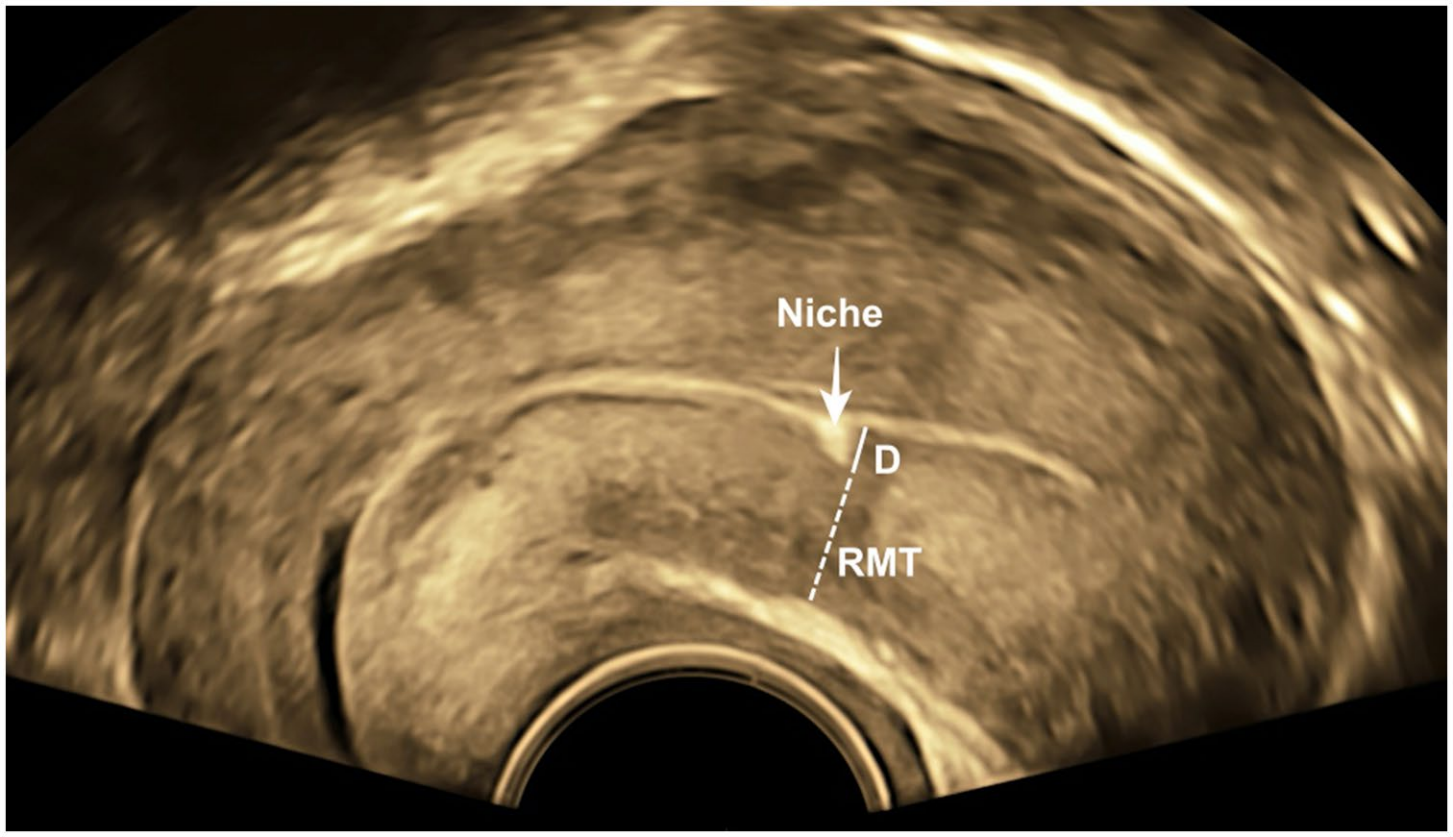
Transvaginal ultrasound with uterine sagittal view demonstrating how RMT ratio was measured. arrow: the myometrial defect in the form of a niche; *D* the depth of the myometrial loss, *RMT* residual myometrial thickness. RMT ratio = RMT × 100/(RMT + D)

This is a prospective cohort study where women with a history of only one CS were recruited 12–24 months postoperatively. Inclusion criteria are age above 18, gestational age at delivery between 24 + 0 and 42 + 0 weeks, elective, unplanned and emergency CS, and signing a consent form. Exclusion criteria were a history of two CSs or more, a history of vertical uterotomy, and a history of additional uterine surgery. The myometrial scar assessment was performed with vaginal ultrasound as shown in Fig. 1. Sonographic volumetric datasets from each patient, where the uterus was completely visualized, were acquired with a 5–13 MHz micro-convex transvaginal transducer, GE RIC6-12-D (Voluson E10, GE Healthcare GmbH, Munich, Germany). The desired planes for evaluating the myometrial defects at the CS scar were acquired with multiplanar views. The residual myometrial thickness (RMT) was measured according to the recommendations of Jordans et al. [9]. The depth of the myometrial loss (D) at the CS scar was measured on the sagittal plane perpendicular to the endometrial line. The degree of myometrial loss was quantified by calculating an RMT ratio as a percentage of RMT to the assumed original pre-cesarean anterior uterine wall thickness. The formula utilized for this calculation is RMT ratio = RMTx100/(RMT + D).

Cutaneous scar assessment was performed according to the MSS. Assessment was carried out by a member of the study team who was not involved with the operation to avoid bias. The MSS evaluates and scores four parameters including scar color (perfect match, slight-, obvious-, or gross- mismatch with surrounding skin), surface shine or appearance (matte or shiny), contour (flush with surrounding skin, slightly indented, hypertrophic or keloid), and distortion (none, mild, moderate or severe). The score for each parameter begins with 1 and increases up to 4 according to the quality of the characteristic. The range of the final score is between 4 and 14 where lower values denote a better healing outcome, while a high total score indicates abnormal scarring [5]. A tabular presentation of MSS is shown in Table 1.

**Table 1:** Manchester Scar Scale (MSS) with the assessed parameters and their corresponding value [5]

MSS parameters	Category	Points
Scar color	Perfect match Slight mismatch Obvious mismatch Gross mismatch	1 2 3 4
Surface shine	Matte Shiny	1 2
Contour	Flush with surrounding skin Slightly indented Hypertrophic Keloid	1 2 3 4
Distortion	None Mild Moderate Severe	1 2 3 4
Outcome	Best Worst	(4– 14)

Spearman’s correlation test was used to assess the correlation between the individual MSS and RMT ratio. Patients were further subdivided into ethnical groups in order to investigate the effect of ethnicity on both MSS score and RMT ratios with the Kruskal–Wallis test. The cut-off value of *p* below 0.05 was considered to be significant.

## Results

**Fig. 2:**
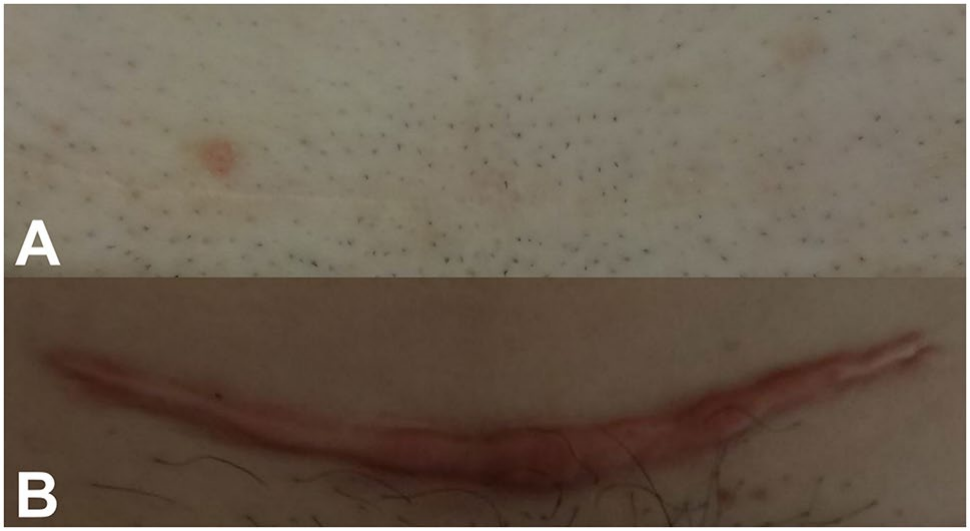
Photographic appearance of Cesarean section laparotomy scar showing **a** perfect healing with MSS 4 and **b** severe scarring with MSS 13

This study included 247 women, with a 2.4% Asian, 3.6% Afro-American, 82% Caucasian and 12% Mediterranean background. The average point in time of recruitment was 18 months postoperatively. The RMT ratio ranged between 11.9 and 100% with a median of 55.8% and an average of 56%. The range of MSS scores for this cohort was between 4 and 13 with a median of 5 and an average of 6 (approximated from 5.82). Figure 2 shows the two extremes of the MSS for this study cohort.

**Fig. 3:**
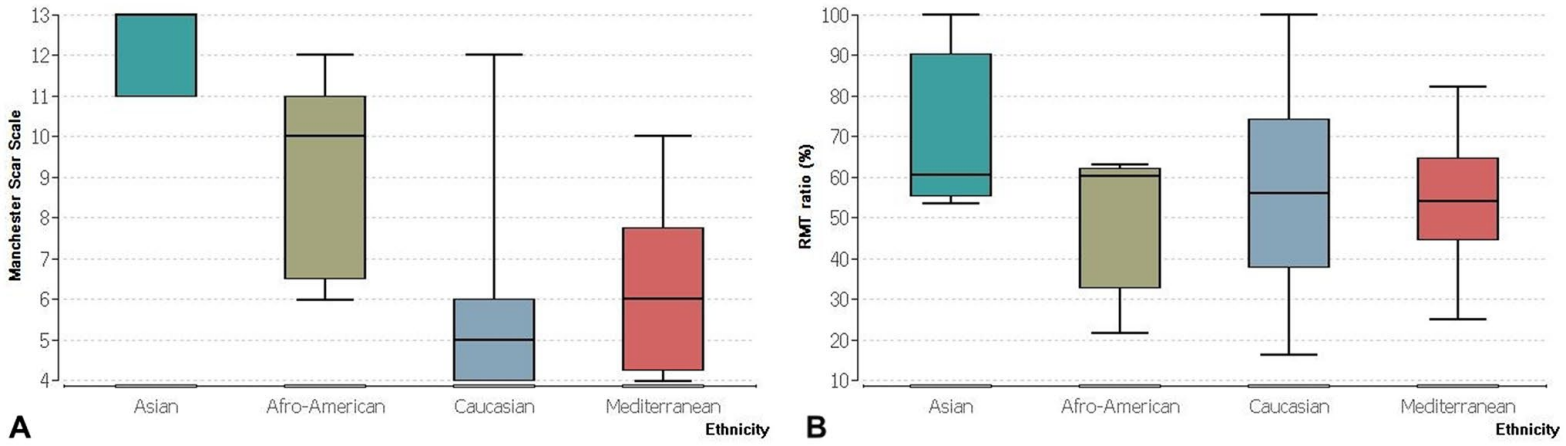
Box plot showing the medians and distribution of **a** MSS and **b** RMT ratio depending on ethnicity

Spearman’s correlation test between MSS and RMT ratio showed a rho of − 0.01 with a *p* value of 0.8. The *p* value remained high between 0.3 and 0.8 and the rho ranged between 0.8 and − 0.8 when the correlation between RMT ratio and MSS was tested within the separate ethnical groups. The Kruskal–Wallis test for the effect of ethnicity on MSS showed an eta^2^ of 0.13 and a *p* value of 0.0002. The effect of ethnicity on the RMT ratio showed an eta^2^ of 0.009 and a *p* value of 0.68. The differences between both the MSS scores and the RMT ratio among the ethnical groups is demonstrated in Fig. 3.

## Discussion

Wound healing in adult humans can invariably cause scarring and subsequently have an adverse effect on both function and cosmetic appearance. The process of wound healing is governed by genetic, immunological, and hormonal factors that lead to tissue regeneration. The phases of exudation, resorption, and regeneration during wound healing are mutual between the cutaneous and serosal wounds and the myometrium is also expected to be associated with skin healing [10]. The characteristics of a cutaneous CS scar have shown an association with intraabdominal adhesions during repeat CS [11]. While the assessment of adhesions is difficult to quantify, objective quantifiable myometrial CS scar assessment is possible in a non-gestational state. Thus, we decided to implement two quantitative methods for assessing intraindividual healing results of both the cutaneous scar and the myometrial scar post-cesarean. A Delphi-based guideline was published in 2019 to standardize the sonographic measurement methodology of CS scars [9]. The measurements of our study were based on this guideline, and in order to account for individual uterine size differences between different women the RMT ratio was utilized [12]. Other studies employed similar methods and decided to assess CS scars with ratios instead of utilizing absolute values. The myometrial thinning at the scar was measured as a deficiency ratio and a ratio above 50% was considered to be severe. Our proposed RMT ratio has an inverse relationship to such a deficiency ratio and a higher RMT ratio reflects better healing [13]. Our data show both median and average RMT ratios of around 56% which contradicts the assumption that a deficiency above 50% is severe.

The diverse variety of surgical techniques involved with CS and the importance of evidence-based practices regarding skin closure have led to increasing research interest for evaluating CS procedures [14]. Surgical wound complications and infections are the most common adverse outcomes after CS with an incidence between 4.9% and 9.8% and collectively represent the most costly complication of CS [15]. In an attempt to define important criteria for assessing wound healing, clinicians rely on both cosmetic and functional parameters [16]. While the MSS is based mainly on cosmetic criteria, its score correlates directly with the histological features of the scar tissue. It is objective, reproducible, valid, uncostly in both time and manpower, and simple to implement [5] Therefore, it is one of the most popular scores among clinicians [17]. Therefore, we opted to implement the MSS for this study also, and our median and averages scores of 5 and 6, respectively, show that this cohort generally exhibited good wound healing. We could have attributed this good healing to the usage of sutures instead of clips for skin closure, but randomized controlled trials have shown otherwise. They conclude that the cosmetic appearance of the cutaneous CS scars is comparable regardless of the skin closure technique. Therefore, a patient’s own healing potential was attributed as the significant influencing factor [2]. It is safe to assume that all wounds in our cohort, both cutaneous and myometrial, had reached their full healing potential due to the average recruitment time of 18 months postoperatively. It is known that cutaneous wounds finish healing within six months and myometrial CS scars reach their healing potential between 6 and 9 months post-cesarean and remain constant up to a following pregnancy [18].

Both myometrial and cutaneous CS scars are the product of wound-healing processes, thus they share similar biologic healing pathways [19]. Transforming growth factor β (TGF-β) plays a pivotal role in the process of wound healing. TGF-β induces the production of collagen fibers and TGF-β overexpression leads to abundance of collagen that results in hypertrophic scars and keloid formation [20]. Altered or reduced expression of TGF-β on the other hand leads to impaired collagen deposition in the scar tissue and results in abnormal scar formation or scar dehiscence of the lower uterine segment [21]. Therefore, we expected to show a negative correlation between MSS and RMT ratio for our patients. Our data were unable to support this theory as the correlation between RMT and MSS showed a statistically insignificant p value of 0.8. Even with further subdivision of the cohort into ethnical groups, a correlation between MSS and RMT could not be demonstrated statistically, which leads us to reject our proposed correlation between the two. Several studies attributed the increased collagen production during healing as the main reason for the association between postoperative high cutaneous MSS values and intraabdominal adhesions [22]. This mechanism does not apply to the myometrial healing in this cohort.

Ethnicity affects cutaneous scar healing significantly. Individuals from Asian, Afro-American, and Mediterranean backgrounds possess the so called ‘ethnic skin’, which is highly susceptible to scarring and keloid formation [23]. This fact is evident in our cohort as Asian, Afro-American, and Mediterranean patients had significantly higher MSS scores compared to Caucasian women and the Kruskal–Wallis results prove this effect with an eta^2^ of 0.13 and a *p* value of 0.0002. The RMT ratio, however, was unaffected by ethnicity and Fig. 3 shows very close RMT ratio medians for the four selected groups. Myometrial healing is a complex process which is not only governed by genotypic, but also phenotypic factors and leads to heterogenous behaviors of myometrial surgical wounds [21]. While several factors can affect scar healing, individual healing potential varies from one person to another and plays a significant role in cutaneous scarring [24]. One limitation to our study is the uneven distribution of the different ethnicities among the cohort which might cause bias.

In conclusion, even though there are differences in the appearance of the cutaneous CS scar between ethnical groups, these scars usually heal with satisfying results. These scars do not represent the myometrial healing after CS and separate uterine sonographic assessment is recommended for future counseling. Ethnicity does not affect the myometrial healing and further research is needed to identify influencing factors.
